# Surgical Management of Massive Metal Bezoar

**DOI:** 10.7759/cureus.12597

**Published:** 2021-01-09

**Authors:** Ahmed M AlMuhsin, Fatima Alsalman, Ahmad Bubshait, Rami O Abu Hajar

**Affiliations:** 1 Department of General Surgery, Security Forces Hospital, Dammam, SAU

**Keywords:** foreign body, metal bezoar, laparotomy, case report

## Abstract

Ingestion of foreign bodies is common within the pediatric population; in adults, it occurs more commonly in those with a psychiatric background. Diagnosis of such cases can be readily made based on plain abdominal X-rays. As reported, many foreign bodies pass through the gastrointestinal tract without complications, obstruction, bleeding, and perforation. The ultimate decision of the best management approach for such cases should be made based on the available expertise as well as the patient’s specific factors. Observation, endoscopic removal, and surgical intervention are all acceptable approaches in cases of metal foreign body ingestion. We report a case of a 29-year-old male patient brought to the emergency department following ingestion of multiple sharp nails. He underwent surgical exploration, which resulted in the retrieval of 73 metallic nails.

## Introduction

Foreign body (FB) ingestion is common within the pediatric age group, and to a lesser extent in adults, especially those with an underlying psychiatric illness [1-3]. These indigestible foreign bodies accumulate within the gastrointestinal tract and are known as bezoars. Based on the FB type and composition, several types of bezoars have been identified [2]. The most common type is phytobezoar, which composes of indigestible vegetable fibers. Pharmacobezoars are formed of indigestible medications. Trichobezoars are mainly composed of hair, and when they extend beyond the stomach, they are known as Rapunzel syndrome. Lactobezoars are seen in infants, and it's mainly formed from undigested milk and mucus. Metal bezoars are the least common type [1, 2]. Ingestion of a massive number of sharp nails is an example of metal bezoars with few cases have been reported in the literature. Those patients usually require intervention as they carry a risk of complications in the form of perforation, bleeding, and obstruction [1-3].

## Case presentation

**Figure 1:**
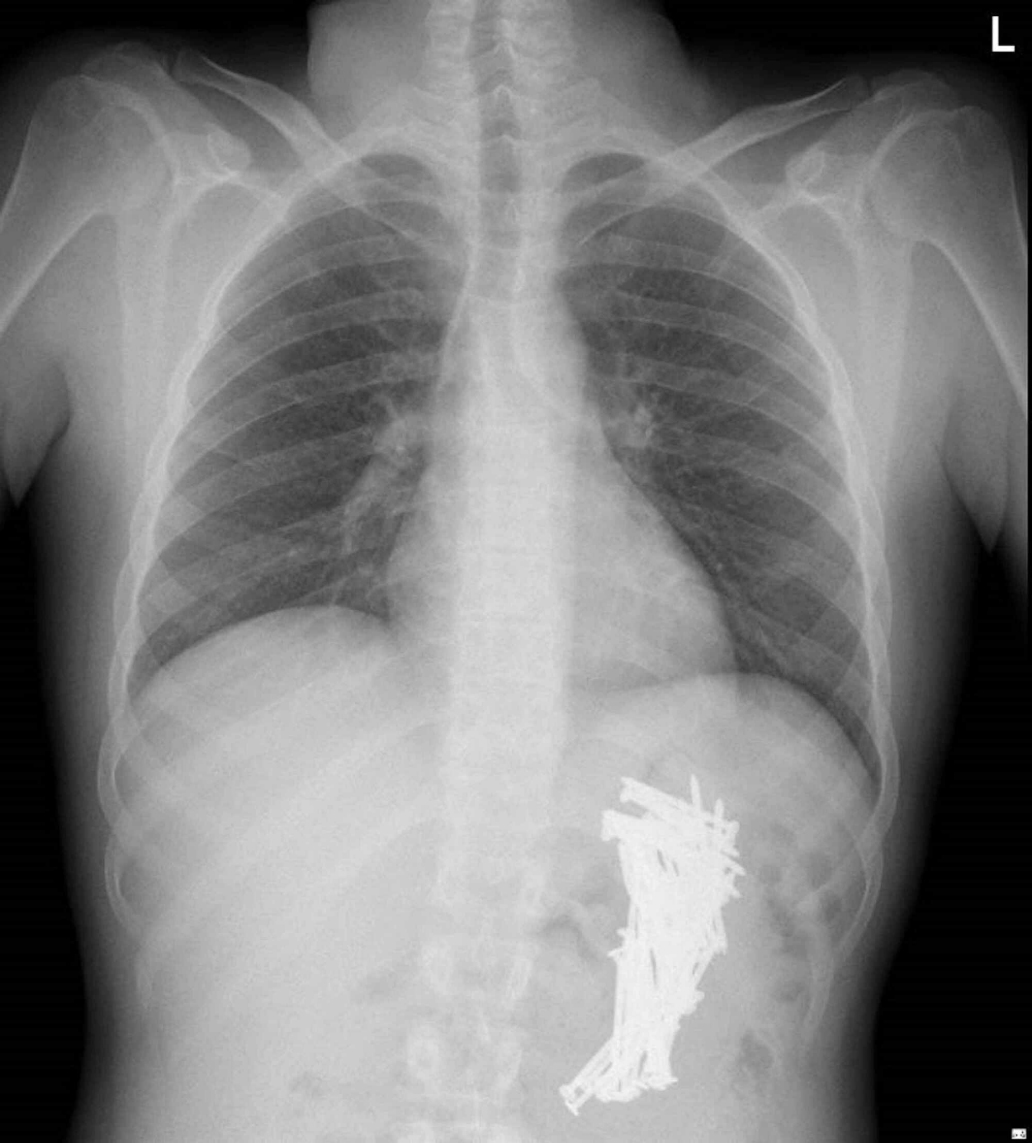
Chest X-ray showing multiple metallic nails within the stomach, with no evidence of air under the diaphragm

**Figure 2:**
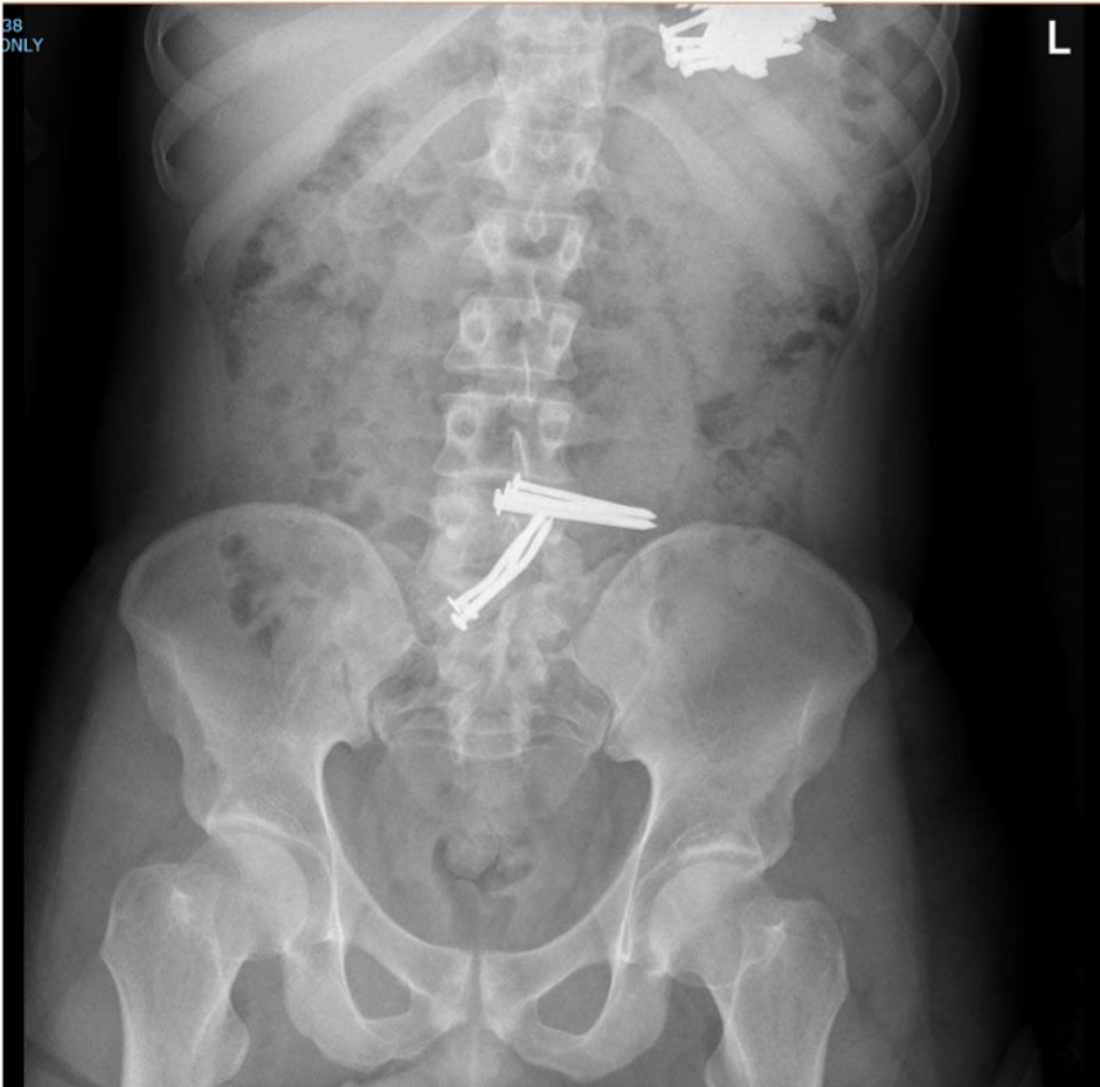
Plain abdominal X-ray showing multiple metallic nails within the stomach and the small bowel

A 29-year-old male patient was brought to the emergency department (ED) by his family after witnessing swallowing multiple sharp metallic nails a day before the presentation. Upon assessment, he was completely asymptomatic. He had no history of abdominal pain, nausea, or vomiting. He didn’t report any changes in his bowel habits. The patient was noted to have some bizarre behaviors indicating poor judgment and low intellectual ability. Additionally, he was believed to have a regular follow-up in a psychiatric institute a couple of years back; however, he was not on any regular medication at the moment of presentation. Moreover, the family reported a history of substance abuse. On examination, his vital signs showed a pulse rate of 78 beats per minute, blood pressure of 117/78 mmHg, a temperature of 36.8°C, and oxygen saturation of 99% on room air. Local examination revealed a soft and lax abdomen with mild tenderness at the epigastric area. Laboratory investigations showed hemoglobin 15 mg/dl (13.5-17.2), white cell count 14.6 C 103/μL (4.0-11.0), neutrophils count 9.27 C 103/μL (2.0-7.0), lymphocytes count 3.64 C 103/μL (1.0-3.0). Chest and abdominal X-rays were obtained and showed multiple metallic nails in the left upper quadrant and lower abdomen, with no evidence of air under the diaphragm (Figures 1-2).

**Figure 3:**
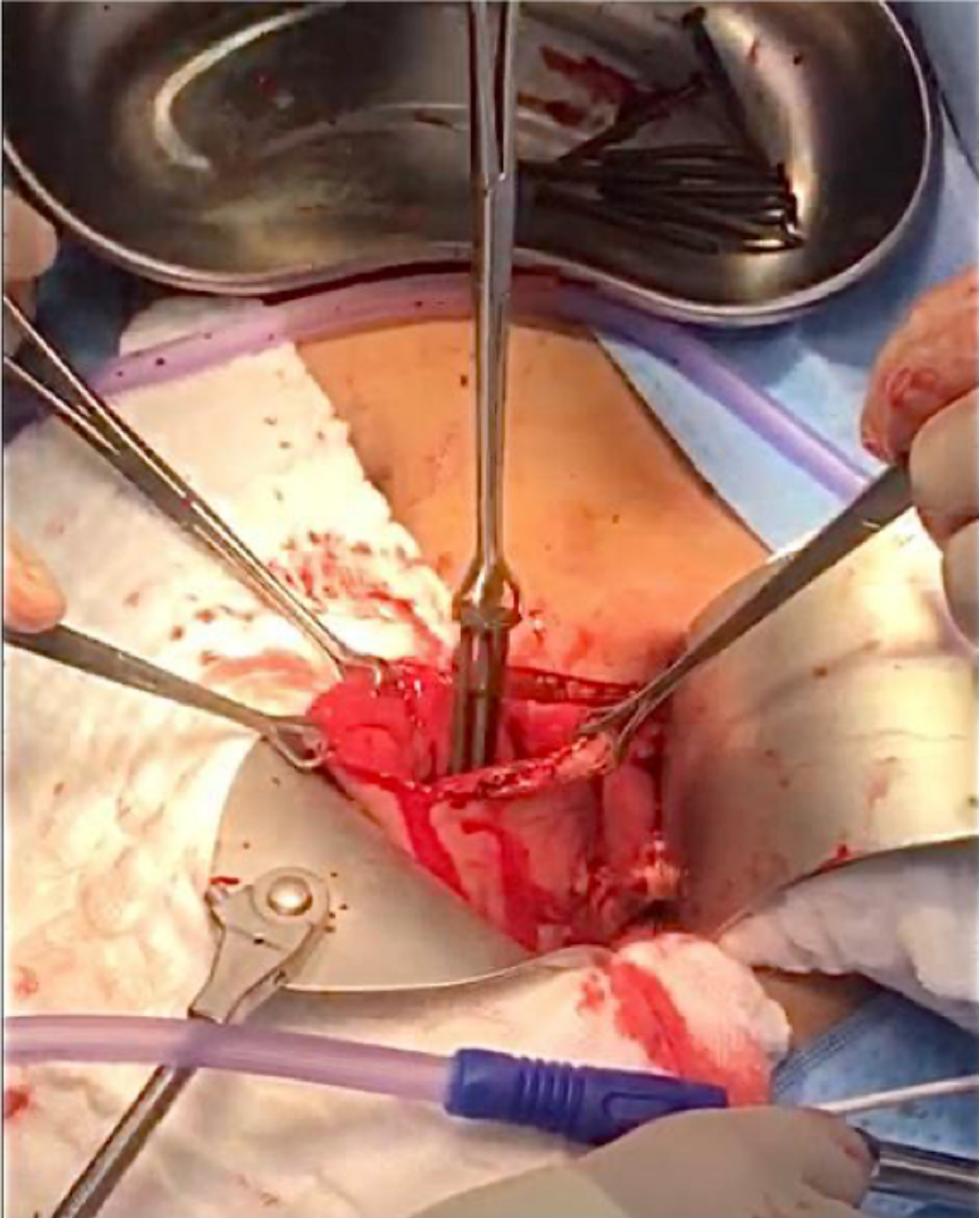
Intraoperative picture showing the stomach with multiple metallic nails being extracted through the gastrostomy

A decision was made to take the patient for an exploratory laparotomy considering the patient’s clinical status and abdominal X-ray findings. Exploration was performed through a midline laparotomy incision, revealing no gross evidence of perforation. A gastrotomy was performed, and a total of 52 nails were extracted from the stomach (Figure 3).

**Figure 4:**
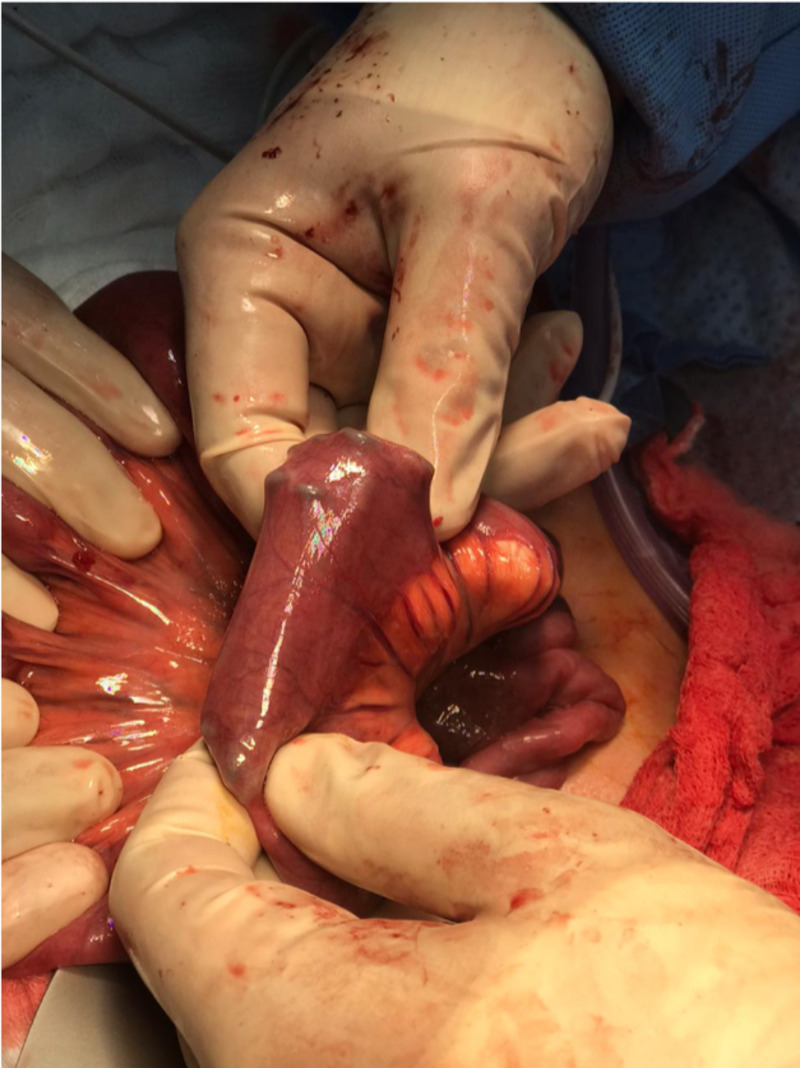
Intraoperative picture showing multiple metallic nails within the terminal ileum

**Figure 5:**
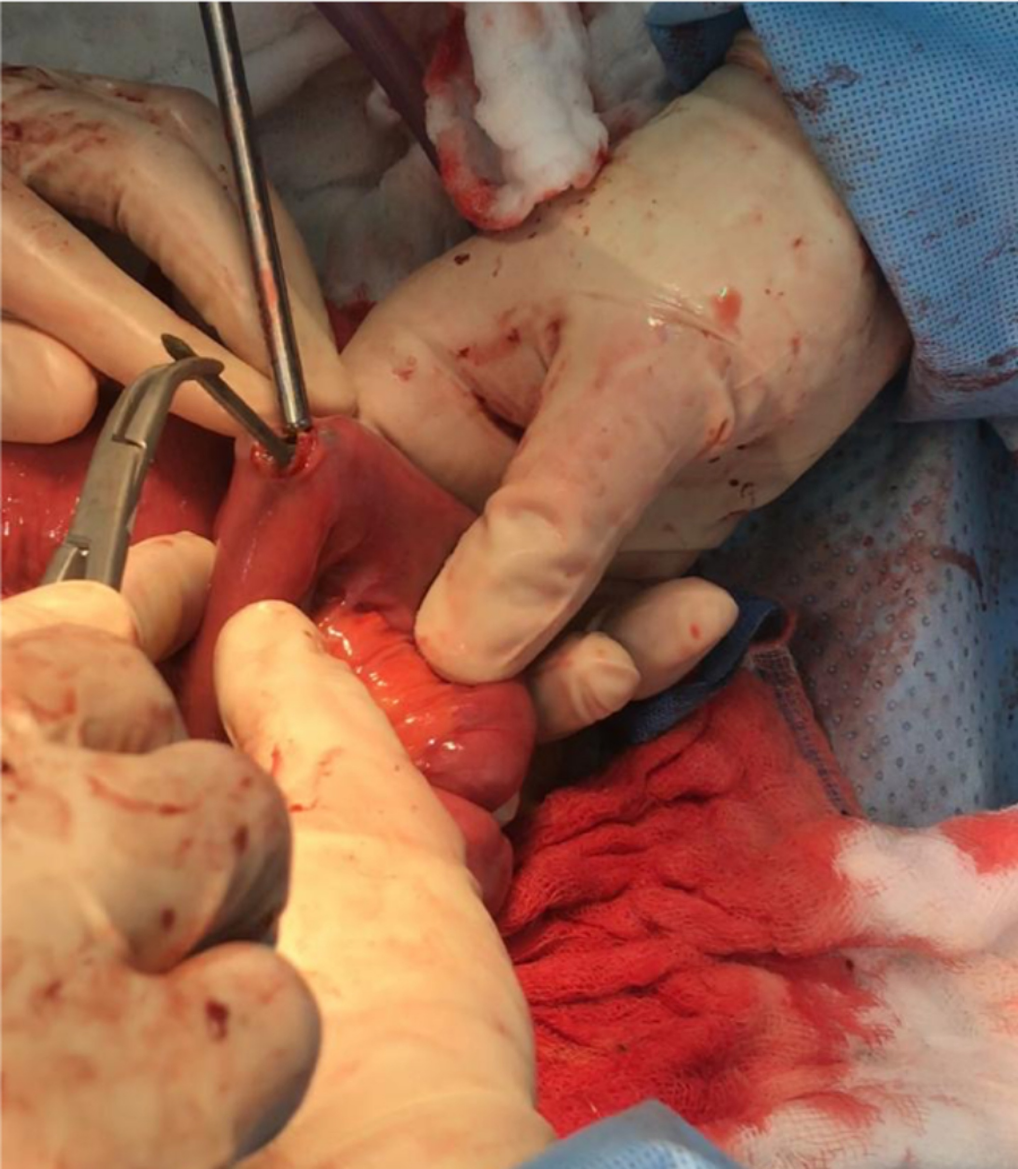
Intraoperative picture showing the terminal ileum with multiple metallic nails being extracted through an enterotomy

The bowel was then examined from the duodenojejunal junction to the ileocecal valve. Multiple nails were palpable at 100 cm proximal to the ileocecal junction. An enterotomy was performed, and 14 nails were extracted (Figures 4-5).

**Figure 6:**
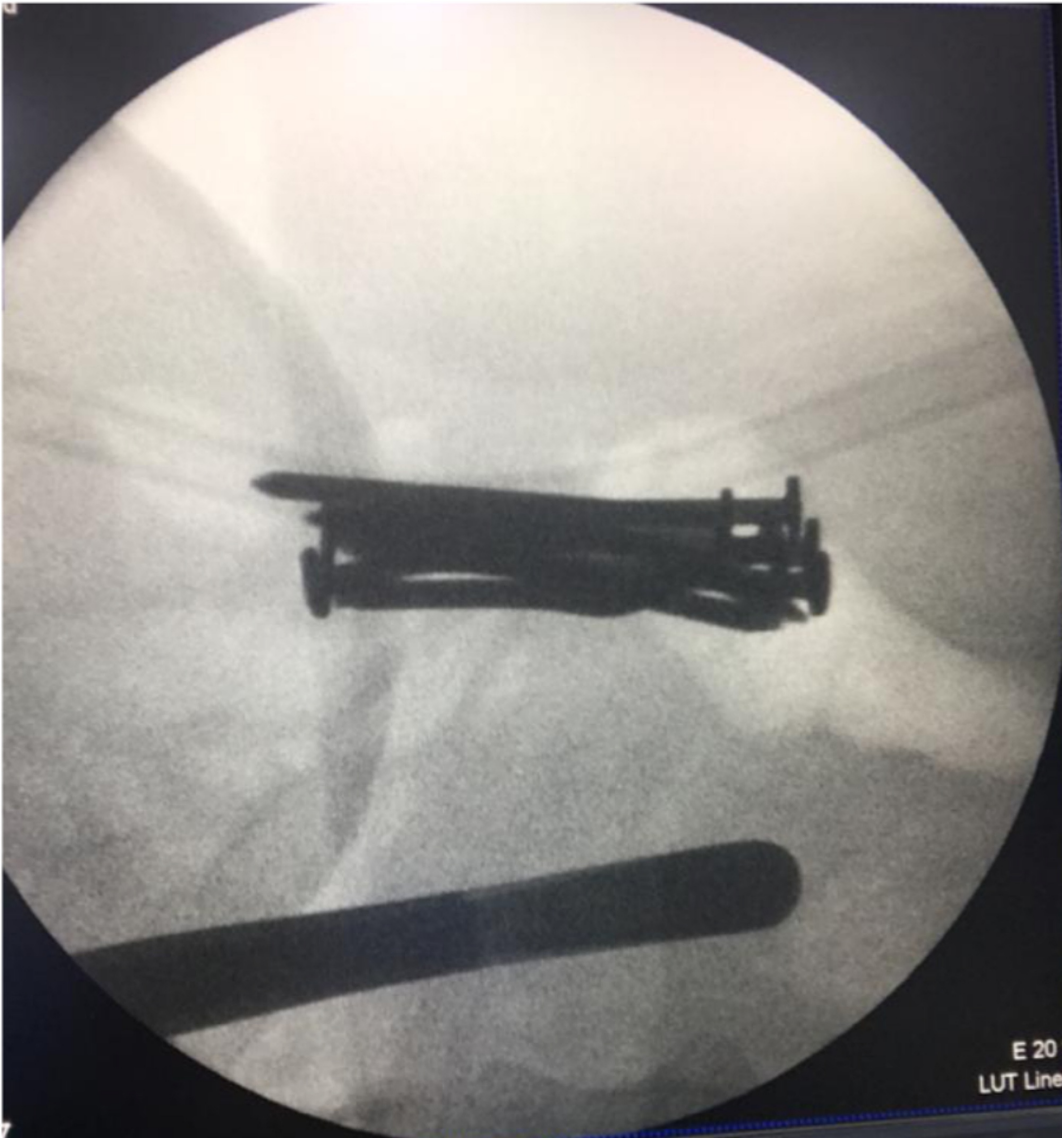
Intraoperative picture demonstrating the use of C-arm fluoroscopy in detection and localization of the foreign bodies within the gastrointestinal tract

The gastrostomy and the enterotomy were closed in two layers using an absorbable suture. After further examination and with the aid of the C-arm fluoroscopy, a cluster of nails was found at the vicinity of the ileocecal junction. Therefore, an appendectomy was performed, and a total of seven nails were extracted through the appendicular stump. Finally, intraoperative C-arm fluoroscopy was performed for assessment and showed no remaining nails within the abdominal cavity (Figure 6).

**Figure 7:**
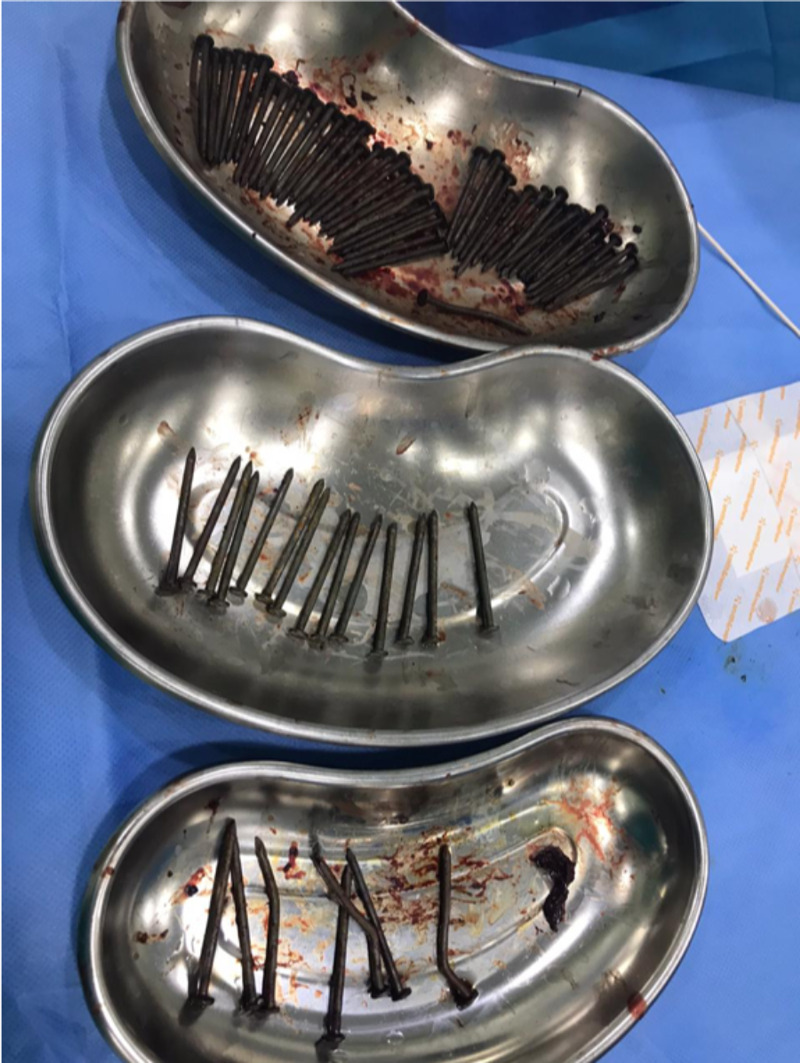
Intraoperative picture showing a total of 73 sharp nails extracted successfully from the gastrointestinal tract

Thus, a total of 73 nails were extracted successfully (Figure 7).

The patient had an uneventful postoperative course. The nasogastric tube was removed on the second postoperative day, at which he started tolerating oral feeding. The patient was discharged home on the ninth postoperative day and was followed in the outpatient department for three weeks. He developed a mild wound infection, for which he was treated with oral antibiotics and regular wound dressing. Psychiatric consultation was sought during the patient’s hospital course. He was started on atypical antipsychotic medication (olanzapine), to which he responded very well. Eventually, he was referred to a specialized psychiatric institute.

## Discussion

The term bezoar is derived from the Arabic term (bezehr) or (bedzher), which goes historically to more than 2000 years ago. Bezoar can be defined as a foreign body resulting from an accumulation of indigestible materials commonly found as a mass within the gastrointestinal tract (GIT) [1, 2]. They are most commonly found in the stomach; however, they can be found in the esophagus, small bowel, and colon. Bezoars are classified according to their composition into phytobezoars, pharmacobezoars, trichobezoars, lactobezoars, and foreign body bezoars [2]. Metal foreign body bezoars are the least common form, with a few cases have been reported in the literature [3].

Although foreign body bezoar occurs in all age groups, pediatric populations represent the vast majority of cases, with a peak incidence between the age of six months and six years [4]. Children are more likely to ingest coins, toys, safety pins, or small batteries. However, adult patients usually present with meat and fish bones, toothpicks, denture parts, nails, pins, and screws as foreign bodies that have been accidentally ingested [5]. Intentional and repeated FB ingestion is encountered principally in adult patients with psychological disorders, intellectual disability, alcohol intoxication, prison inmates, and for drug smuggling [4, 5].

Although most foreign bodies pass spontaneously without intervention, there is a risk of obstruction, impaction, hemorrhage, or perforation, particularly at the sites of acute angulation or narrowing in the gastrointestinal tract. Among these sites, the narrowing at the level of the cricopharyngeus muscle and the ileocecal valve is the most significant. Moreover, the esophagus is considered the most frequent obstruction site in the gastrointestinal tract due to either a physiological or pathological narrowing. Once the objects pass beyond the esophagus, the vast majority will probably exit the GI tract without complications [5]. Consequently, if no complications manifest, the clinical signs will be minimal or even absent [6]. Symptoms vary and include anorexia, nausea, bloating, early satiety, dyspepsia, dysphagia, odynophagia, malaise, fatigability, and headaches [6, 7].

Approaches to cases with FBs ingestion should be tailored according to the patient’s history and clinical examination. Furthermore, prompt management of certain cases should not be delayed for unnecessary investigations [5, 8]. Biplane plain radiographs have been recommended as the initial modality for diagnosis, as they can not only identify most of FBs in the gastrointestinal tract but can also aid in detecting subsequent complications [7]. However, they fail to identify radiolucent FBs, such as fish or meat bone, plastic, and wood [4]. Additionally, it has a false-negative rate of 0.5% to 47%, and a 20% false-positive rate [9, 10]. Computed tomography (CT) scan is another modality for diagnosis with higher sensitivity, especially with a 3-dimensional reconstruction [4, 7]. Generally, it is accepted that barium studies should not be used in such cases, as they may delay and complicate endoscopic evaluation and retrieval of FBs [10]. Endoscopy is considered the most accurate diagnostic modality in suspected FB ingestions [10]. The metal detector has been encouraged by many authors as an accurate tool for localization of ingested metallic FBs; however, its application is limited due to restricted availability and limited application in adults [11]. C-arm fluoroscopy is extremely useful in identifying and localizing metallic foreign bodies intraoperatively [12]. In our case, intraoperative fluoroscopy has been used to localize missed FBs, avoid unnecessary exploration, and avoid subsequent complications.

Multiple perspectives should be taken into account when managing FB ingestion patients, including patient’s age, clinical condition, FB features (length, size, number, type of material), anatomical site of ingested FB, and the expertise of the endoscopist. Although many foreign bodies ingested will pass spontaneously following conservative management, up to 20% of cases will require endoscopic retrieval, and rarely (1%) will require surgical intervention [5]. However, different groups from China, Korea, and Italy reported higher figures of FBs that had been removed endoscopically (80-90%), with other recent studies reporting the need for surgical intervention in up to 16% of cases [4, 13]. Accordingly, objects greater than 2 to 2.5 cm in diameter are less likely to pass through the pylorus or ileocecal valve, and objects longer than 5-6 cm are unlikely to pass through the duodenal sweep [4, 8]. Thus, these FBs require endoscopic removal. Similarly, this applies to smaller objects that failed to bypass the pylorus after three to four weeks. As the surgical intervention is warranted in the setting of complications and failure of endoscopic retrieval, it should be considered for FBs located distal to the duodenum that showed no progression for one week and for sharp objects that failed to progress after three days [4]. Furthermore, it has been recommended that sharp objects like metallic nails should be removed even if the patient did not develop complications, since the morbidity and mortality are higher with these objects, particularly when it is associated with a large number of sharp objects as in the reported case [14]. Conversely, a wait-and-watch policy for a massive number of sharp FBs is an acceptable approach [5]. Hendry et al. reported successful conservative treatment for a patient who had ingested 20 razor blades [15]. Vats et al. also reported successful non-operative management for a patient presenting with abdominal pain following ingestion of nine sharp nails [5].

Appendicostomy has been reported as the safest approach for the extraction of foreign bodies located in the vicinity of the ileocecal junction [16]. Mohammed et al. reported a similar approach to extract metallic nails impacted in the cecum [17]. However, we performed appendectomy and extracted the impacted FBs through the appendicular stump successfully.

## Conclusions

The management of cases with massive FB ingestion should be individualized taking into account the patient’s clinical status and the anatomical site of the FB. Early surgical intervention can be considered an appropriate approach in such cases, as it may avoid subsequent complications such as perforation, hence avoiding unnecessary bowel resection. Psychiatric counseling is crucial in these situations. It will elicit the psychological background, or adjusting the maintenance medications in known cases, therefore preventing recurrence and relapse.
